# Pathology of atherosclerotic coronary artery disease in the young Indian population

**DOI:** 10.1080/20961790.2019.1592315

**Published:** 2019-05-16

**Authors:** Pradeep Vaideeswar, Shashank Tyagi, Saranya Singaravel

**Affiliations:** a Department of Pathology (Cardiovascular & Thoracic Division), Seth GS Medical College & KEM Hospital, Mumbai, India;; b Department of Forensic Medicine & Toxicology, Seth GS Medical College & KEM Hospital, Mumbai, India

**Keywords:** Forensic sciences, forensic pathology, ischemic heart disease, coronary atherosclerosis, young adults, sudden cardiac death

## Abstract

Atherosclerotic coronary artery disease (CAD) is of great concern in young adults because of its potential to cause great incapacitation. This arena of cardiology has gained importance in South Asian countries, particularly India due to increased prevalence that is related to traditional risk factors, altered life styles and inherent risk factors. In this study, we sought to evaluate, at autopsy, the pathology of atherosclerotic CAD in young patients with ischemic heart disease (IHD). A 10-year retrospective autopsy-based study was carried out in a large tertiary-care centre and patients aged ≤45 years with IHD were selected. Out of 545 autopsied cases of IHD, 95 patients (17.4%) were young. Among these 95 patients, 84 (88.4%) had IHD related to atherosclerotic CAD; the youngest patient was 18 years old. Predictably there was sole involvement of left anterior descending artery and the presence of fibrous plaques. Irrespective of the plaque morphology, the commonest complication was thrombosis produced by plaque erosion seen in 36.9% of patients. Acute coronary insufficiency was noted in 52 patients (61.9%), while healed infarctions were surprisingly noted in 28 patients (33.3%). Screening for IHD in the young population may help to improve prognosis by detecting subclinical disease, although more studies are necessary to establish reference limits for this young population. Additional research must also focus on treatment concerns that are specific to young patients.

## Introduction

Non-communicable diseases are disorders of long duration and slow progression. Important among them are cardiovascular diseases (CVD), cancers, chronic respiratory diseases and diabetes mellitus (DM). Collectively, besides producing morbidity and disability, they are the leading causes of global mortality. A similar scenario exists in India, where CVDs account for nearly 30% of such disorders [1]. The underlying pathology in most patients in the Indian sub-continent is atherosclerosis, whose progression and/or acceleration is proportional to the traditional risk factors, altered life styles as well as inherent risk factors [1]. By and large, this atherosclerotic process culminates in ischemic heart disease (IHD) and a worrisome fact is an increasing incidence of IHD in the young population (≤45 years of age) [2]. Furthermore, the initial manifestation of IHD in these young persons may be in the form of sudden cardiac death (SCD). In this study, we sought to evaluate, at autopsy, the pathology of atherosclerotic coronary artery disease (CAD) in young patients with IHD.

## Materials and methods

**Figure 1.**
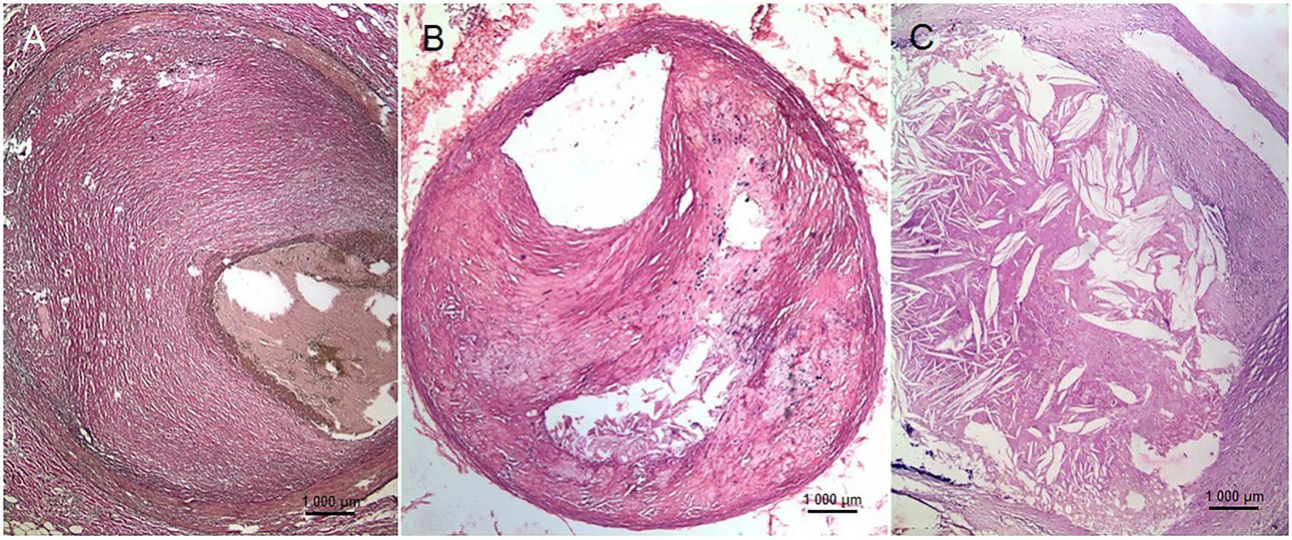
(A) Fibrous plaque, which is fibrocellular and also rich in proteoglycans (Elastic van Gieson, ×100); (B) Fibro-fatty plaque, composed of an equal proportion of fibro-cellular and lipid elements (HE, ×100); (C) Fatty plaque, comprising a large lipid-rich core separated from the lumen by a thin fibrous cap. The lipid material may in the form of collections of foamy macrophages and/or extra-cellular lipid material (HE, ×100).

In a 10-year retrospective autopsy-based study (2008–2017) carried out in a large tertiary-care centre (catering to largely the lower socio-economic group), all cases of IHD that caused death or was identified as a co-morbid condition were reviewed and patients aged ≤45 years were selected for further analyses. A complete autopsy examination was carried out in all the cases according to standard uniform guidelines practicing in our country. SCD was defined as an unexpected natural death occurring within 1 h after onset of symptoms in an apparently healthy subject or in one whose disease was not so severe as to predict an abrupt outcome [3]. When un-witnessed, it referred to death of an individual within 24 h after being seen alive and in a normal state of health. It also included patients with sudden rapid deterioration, who would have an illness, not always expected to cause death. The major epicardial arteries had been studied by serial cross-sections at an interval of 0.5 cm and the sites of maximal narrowing or occlusion were taken for histopathology with inclusion of arterial segments, proximal and distal to stenosis/occlusion as well. The plaques were simplistically categorized as fibrous, fibro-fatty and fatty (Figure 1). The degree of stenosis was classified into three categories: less than 50%; equal to or more than 50%, but less than 75%, and equal to or greater than 75% (critical stenosis). The fibrous plaques showed smooth muscle proliferation in a background of collagen and proteoglycans, while the fatty plaques were composed a large lipid-rich core separated from the lumen by a thin fibrous cap. The lipid component was formed of collections of foamy macrophages and/or extra-cellular lipid material. The fibro-fatty plaques consisted of an equal proportion of fibro-cellular and lipid elements. Presence of complications and other histological changes such as calcification and inflammation were also noted. Depending on the morphology of the myocardium, IHD was classified as acute coronary insufficiency (ACI, critical coronary stenosis devoid of visible ischaemic changes), acute myocardial infarction (AMI), acute-on-chronic myocardial infarction (A-CMI) and chronic myocardial infarction (CMI). These were correlated with the clinical features, risk factors and investigations (obtained from the inquest papers and/or hospital records) in two age groups ≤30 years and from 31–45 years. The study was approved by the Institutional Ethics Committee-II, Seth GS Medical College and KEM Hospital, Mumbai for years 2014–2017.

## Results

In a span of 10 years, there were 545 autopsied cases of IHD; 95 patients (17.4%) were young (≤45 years). In 11 of these patients, the IHD had been related to abnormal coronary ostial localizations, coronary vasculitis, vasospasm, left ventricular hypertrophy and hypercoagulability. The remaining 84 patients (15.4%) had atherosclerotic CAD and were further analysed; only 13 were women (15.5%). The youngest patient was 18 years old.

### Patients ≤30 years

There were 14 patients in this group (16.7%), including 12 males and 2 females with an age range of 18 to 30 years (mean age of 26.8 years); only three patients had hospital admissions for a period of 2–5 days. Dyspnoea and/or chest pain were the presenting symptoms in nine patients. Two patients apparently “dropped dead”, one of whom was undergoing a pleural tapping. Two others developed sudden-onset unconsciousness, with preceding generalized tonic clonic convulsions in one of them. Another patient, an 18-year-old girl with systemic lupus erythematosus (SLE), had been admitted for acute pancreatitis. Hypertension had been present in two other patients. The other risk factors and autopsy findings (if any) are enlisted in Table 1.

**Table 1.** Risk factors and autopsy findings in patients ≤ 30 years (*N* = 14).

Risk factors	Autopsy findings – cardiac	Autopsy findings – non-cardiac
Hypertension (*n* = 2) Diabetes mellitus (*n* = 1)Systemic lupus erythematosus (*n* = 1)	–	Respiratory bronchiolitis (*n* = 1) Pulmonary tuberculosis (*n* = 1) Lupus pneumonitis (*n* = 1) Alcoholic steatosis (*n* = 1) Nodular regenerative hyperplasia (*n* = 1) Acute pancreatitis (*n* = 1)

**Table 2.** Pattern of IHD and coronary atherosclerosis in patients ≤30 years (*N* = 14).

Type of IHD		Type of plaque	
	Fibrous	Fibro-fatty	Fatty
ACI (*n* = 12)	Uncomplicated (*n* = 1) Erosion with fresh thrombus (*n* = 2)	Uncomplicated (*n* = 3) Erosion with fresh thrombus (*n* = 4)	Erosion with fresh thrombus (*n* = 2)
CMI (*n* = 2)	Erosion with organized thrombus (*n* = 1)	Uncomplicated (*n* = 1)	–

IHD: ischemic heart disease; ACI: acute coronary insufficiency; CMI: chronic myocardial infarction.

The heart weights ranged from 220 to 350 g (mean weight of 277.9 g). In all these patients, the left anterior descending artery (LAD) was involved by atherosclerosis (mean length of 1.3 cm). Majority of the patients (12 patients) had ACI (Table 2). This was related to critical atherosclerotic stenosis in four patients, while erosion with occlusive thrombus was seen in eight patients. They occurred in plaques, which had produced critical (75% in five arteries) or non-critical stenosis (50% in three arteries). The healed infarction in two patients showed fibro-fatty plaque with critical stenosis and an eroded fibrous plaque with organized thrombus, respectively.

**Table 3.** Risk factors and autopsy findings in patients 31 to 45 years (*N* = 70).

Risk factors	Autopsy findings – cardiac	Autopsy findings – non-cardiac
Hypertension (*n* = 22) Diabetes mellitus (*n* = 15) Chronic alcoholism (*n* = 11) Smoking (*n* = 5) Obesity (*n* = 1) Systemic lupus erythematosus (*n* = 1)	Patent foramen ovale (*n* = 1) Secundum atrial septal defect (*n* = 1) Rheumatic heart disease (*n* = 1) Arrhythmogenic cardiomyopathy (*n* = 1)	Meningioma (*n* = 1); Pulmonary hemorrhage (*n* = 1) Respiratory bronchiolitis (*n* = 1); Pulmonary tuberculosis (*n* = 1); Pulmonary sarcoidosis (*n* = 3); Invasive aspergillosis (*n* = 1); Lupus pneumonitis (*n* = 1); Bronchiectasis (*n* = 1); Primary pulmonary hypertension (*n* = 1); Intestinal gangrene (*n* = 6); Alcoholic steatosis (*n* = 6); Alcoholic cirrhosis (*n* = 2); Acute pancreatitis (*n* = 5); Acute tubular necrosis (*n* = 1); Renal infarction (*n* = 1); Acute pyelonephritis (*n* = 2); Crescentic glomerulonephritis (*n* = 1); Lupus nephritis (*n* = 1); Focal segmental glomerulosclerosis (*n* = 2); Diabetic nephropathy (*n* = 4); Benign nephrosclerosis (*n* = 3); Splenic infarction (*n* = 1)

**Table 4.** Pattern of IHD and coronary atherosclerosis in patients 31 to 45 years (*N* = 70).

Type of IHD		Type of plaque	
	Fibrous	Fibro-fatty	Fatty
ACI (*n* = 40)	Uncomplicated (*n* = 12) (anomalous origin of left anterior descending artery from right coronary (*n* = 1)) Erosion with fresh thrombus (*n* = 4) Erosion with organized thrombus (*n* = 4) Rupture with fresh thrombus (*n* = 1)	Uncomplicated (*n* = 6) Intra-plaque haemorrhage (*n* = 1) Erosion with fresh thrombus (*n* = 6) Erosion with recanalized thrombus (*n* = 1)	Uncomplicated (*n* = 3) Erosion with fresh thrombus (*n* = 1) Rupture with fresh thrombus (*n* = 1)
AMI (*n* = 4)	Uncomplicated (*n* = 1)	Uncomplicated (*n* = 1) Erosion with fresh thrombus (*n* = 1)	Rupture with fresh thrombus (*n* = 1)
A-CMI (*n* = 5)	Uncomplicated (*n* = 2) Erosion with organized thrombus (*n* = 1)	Erosion with fresh thrombus (*n* = 2)	–
CMI (*n* = 21)	Uncomplicated (*n* = 4) Erosion with fresh/organized thrombus (*n* = 1) Erosion with fresh/recanalized thrombus (*n* = 1) Erosion with organized thrombus (*n* = 3)	Uncomplicated (*n* = 4) Erosion with fresh thrombus (*n* = 2) Erosion with organized thrombus (*n* = 4)	Uncomplicated (*n* = 1) Rupture with fresh/organized thrombus (*n* = 1)

IHD: ischemic heart disease; ACI: acute coronary insufficiency; AMI: acute myocardial infarction; A-CMI: acute-on-chronic myocardial infarction; CMI: chronic myocardial infarction.

### Patients 31 to ≤45 years

Among 70 patients in this group, there were only 11 women (15.7%); mean age was 39.5 years. Dyspnoea with or without chest pain were the presenting symptoms in 33 patients. Ten patients were brought in an unconscious state, while three others collapsed during exertional activities. A 34-year-old woman developed cardiac arrest after administration of general anaesthesia for obstructed labour. Vomiting had been the only symptom in three patients. The remaining 20 patients had been admitted for acute abdomen, acute febrile illness, peripheral vascular disease and limb cellulitis, followed by sudden clinical deterioration. Hypertension and/or diabetes mellitus were present in 32 patients with a past history of IHD in four and cerebrovascular accident in one. The other risk factors and autopsy findings (if any) are enlisted in Table 3.

**Figure 2.**
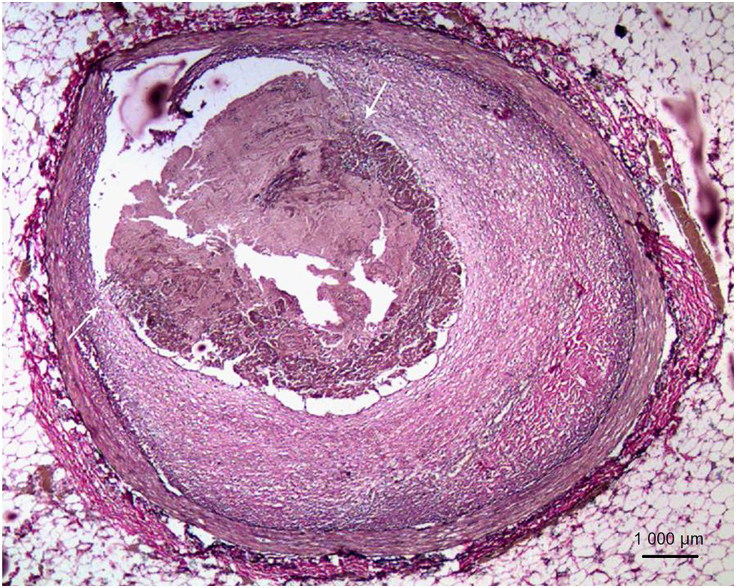
Fibrous plaque with fresh occlusive thrombus. Note adherence at both shoulders (arrows, Elastic van Gieson, ×100).

The heart weights ranged from 180 to 540 g (mean weight of 324.1 g). Single arterial affection was present in 59 hearts (84.3%) and involved the LAD artery in most cases (43 hearts, 72.9%; mean length of 1.1 cm); two or more epicardial arteries were affected in the remaining 16 cases (11.4%). One patient also had an anomalous origin of the LAD from the right coronary. The plaques had been fibrous in 34 (48.6%), fibro-fatty in 28 (40.0%) and fatty in eight patients (11.4%) (Table 4). Critical atherosclerotic stenosis was present in the arteries in 56 hearts (80.0%). ACI, observed in 40 patients, resulted from mere critical stenosis (21 arteries), from thrombotic occlusions related to erosions (16 plaques, Figure 2) or ruptures (two plaques) and intra-plaque haemorrhage (one fibrofatty plaque). AMI and A-CMI were seen in four and five patients, respectively. They were seen with critical stenosis (two arteries) and thrombotic occlusions (five arteries); in two patients, the plaques were found to have produced non-critical stenosis despite presence of infarctions. There were a good number of patients with healed infarctions, 21 (30.0%), related to critical stenosis (nine arteries) and stenosis with thrombotic complications (12 arteries).

Associated calcification in the atherosclerotic plaques was present mainly towards the basal aspects in eight arteries. There was inflammatory infiltrate in the plaque and the adjoining media in 24 arteries, while associated adventitial fibrosis and inflammation was present in 21 arteries. All these changes were prominently seen with fibro-fatty or fatty plaques.

## Discussion

In young adults in general aged 18 to 45 years [4], CVD manifest as both congenital and acquired disorders [5]. The cardiac abnormalities can result in SCD, a devastating event for the family as well as from the societal perspective. We reported a cohort of 84 young individuals who showed features of IHD at autopsy due to coronary arterial atherosclerosis. These cases accounted for about 15.4% of all autopsied cases of IHD, causing SCD; 16.7% were below the age of 30 years. However, this may not represent the true incidence as many of them had not been submitted for a detailed pathological examination, which is extremely essential in young SCDs [6].

The process of atherosclerosis has been known since times immemorial [7] and it has come a long way from being called a “degenerative and proliferative” intimal disease to a chronic inflammatory response to multi-factorial endothelial injury [8]. In general, there seems to be a global rise in the incidence and prevalence of atherosclerotic diseases. But, a premature and rapidly progressive (“malignant”) involvement of the coronary arteries occurs in South Asia, particularly India (15.4% in our series), and this is seen not only in individuals settled in that region but also in the immigrant population, with a lower incidence of less than 3% seen in the Western population [9]. Predictably, males (84.5%) outnumbered women (15.5%). Hypertension, DM, smoking, dyslipidemia, obesity, sedentary life style and positive family history continue to be the traditional risk factors [4, 9]. Hypertension and/or DM was noted in 40.5%, while a history of smoking was documented in only five patients. These were often present in patients over 30 years of age. Interestingly, hypertension was made as a post-mortem diagnosis in five patients on the basis of concentric left ventricular hypertrophy and accompanying early nephrosclerosis changes, indicating a sub-clinical condition. The other non-traditional factors include polymorphisms of genes coding for molecules involved in the lipid metabolism, altered dietary habits, acute phase reactants, micro-organisms, endocrinal disorders, collagen vascular disorders and excessive alcoholic intake [4, 9, 10]. Substances of abuse also predispose to IHD in young patients, due to not only vasopasm but also premature atherosclerosis and its complications [11], but we did not have any such case in this cohort of patients. Among the 13 women in this study, five of them were pregnant with presence of pregnancy induced hypertension in four. Pregnancy is increasingly being recognized as a cause of cardiovascular and metabolic stress [12]. It is also important to remember the psycho-biological aspects, which do not necessarily fall into the purview of the health-care systems [13]. These social determinants can lead to stress, which fosters unhealthy habits and neuroendocrine perturbations with alterations in plaque morphology [14].

Inflammatory cells have always been part and parcel of atherosclerosis. Through release of cytokines, they produce modification, progression and subsequent complications, inducing a constellation of symptoms in the patient. Much prominence has been given to the accumulations of lipids. However, such fatty plaques were noted in only 10 of the arterial segments (12%) studied, which reiterates the fact that lipid-rich plaques are seen in older patients [15]. In the remaining 88%, the plaques were either fibrous or fibro-fatty with little calcification. In concordance with literature [16–18], single artery involvement (73 cases, 86.9%) and involvement of the left anterior descending artery (57 cases, 67%) was also noted in our series. Thrombotic occlusion was seen in 43 arteries (51.2%), and fibrous plaques were involved in 18 of these arteries (41.9%). This is in contrast to the study by Corrado et al [19], where only 27% of the arteries had thrombosis, particularly in non-fibrous plaques. Majority of the plaques (36.9%) had thrombotic occlusion, related to plaque erosion, which does not necessarily rely on the traditional risk factors for its pathogenesis. The erosions are characterized by denudation or disruption of the endothelium that may be related to the haemodynamics of blood flow in the region of the plaque or through cytokines released by T-lymphocytes, macrophages and granulocytes; the latter appears to be aided by the proteoglycan-rich plaques, frequently seen in the young [20]. An interesting finding noted was the presence of healed infarcts in nearly one-third of the young patients (mostly above 30 years of age). IHD had been diagnosed in only four of these patients. This indicates that in the remaining patients, the symptoms could have been ignored or attributed to some other ailment, particularly acid-peptic disease.

This study reiterates the necessity of a definitive protocol to be setup for detailed postmortem examination since significant atherosclerosis can lead to sudden deaths even in the young population. Such a meticulous postmortem coupled with histo-pathological examination minimizes the risk of autopsy being negative or obscure one. The study can be of great help in convincing and counselling bereaved relatives of deceased about cause and manner of death, so that the subtle yet alarming signs and symptoms are not ignored. A strategy for screening and optimal medical therapy can also be worked out in the near future.
